# Reactive oxygen species and aldehyde dehydrogenase 1A as prognosis and theragnostic biomarker in acute myeloid leukaemia patients

**DOI:** 10.1111/jcmm.70011

**Published:** 2024-10-11

**Authors:** G. Venton, J. Colle, A. Tichadou, J. Quessada, C. Baier, Y. Labiad, M. Perez, L. De Lassus, M. Loosveld, I. Arnoux, N. Abbou, I. Ceylan, G. Martin, R. Costello

**Affiliations:** ^1^ APHM, Hôpital de la Conception, Service d'Hématologie et Thérapie Cellulaire Marseille France; ^2^ TAGC‐Theories and Approaches of Genomic Complexity Aix Marseille University Marseille France; ^3^ APHM, Hôpital La Timone, Laboratoire d'Hématologie Marseille France; ^4^ Advanced BioDesign Lyon France; ^5^ CRCM Inserm UMR1068, CNRS UMR7258, Aix Marseille Université U105, Institut Paoli Calmettes Marseille France; ^6^ APHM, Hopital La Timone, Service d'Oncobiologie, Plateforme M2GM, Hopital de la Timone Marseille France; ^7^ Aix‐Marseille Univ, INSERM, INRAE, C2VN, Laboratory of Haematology, CRB Assistance Publique‐Hôpitaux de Marseille, HemoVasc (CRB AP‐HM HemoVasc) Marseille France

## Abstract

Acute myeloid leukaemia (AML) remains a major unmet medical, despite recent progress in targeted molecular therapies. One aspect of leukaemic cell resistance to chemotherapy is the development of clones with increased capacity to respond to cellular stress and the production of reactive oxygen species (ROS), thanks in particular to a high aldehyde dehydrogenases (ALDH) 1A1/2 activity. At diagnosis, ROS level and ALDH1A1/2 activity in AML patients BM are correlated with the different ELN 2022 prognostic groups and overall survival (OS). A significant lower ALDH1A1/2 activity in BM was observed in the favourable ELN2022 subgroup compared to the intermediate and adverse group (*p* < 0.01). In the same way, the ROS levels were significantly lower in the favourable ELN 2022 subgroup compared to the intermediate group (*p* < 0.0001) and adverse group (*p* < 0.0002). ROS^high^ AML patients had a significantly lower median overall survival (OS) (8.2 months) than ROS^low^ patients (24.6 months) (*p* = 0.0368). After first‐line therapy, a significant increase of ROS level (*p* = 0.015) and ALDH1A1/2 activity (0 = 0.0273) in leukaemic blasts was observed, especially in the refractory ones. ABD‐3001, a competitive and irreversible inhibitor of ALDHs 1 and 3, can in vitro inhibit the proliferation of patient‐derived leukaemic cells in accordance with redox balance. In multivariate analysis, ROS level was the most significant (*p* < 0.05) and the strongest predictive factor for the sensitivity of cells to ABD‐3001. The safety profile of ABD‐3001 is currently being assessed through the first inhuman multicenter phase 1 clinical trial “ODYSSEY” (NCT05601726) for patients with relapsed AML.

## 1 INTRODUCTION

Acute myeloid leukaemia (AML) is the most common acute leukaemia in adults, with a median age of 69 years. Most patients with AML achieve complete remission (CR) after standard induction chemotherapy.^1^ However, leukaemic cells develop rapid resistance to the different therapies due to tumour heterogeneity (leading to clonal drift) and/or insensitivity of leukaemic stem cells (LSC) to conventional therapies.^2^ Therefore, the majority of AML patients subsequently relapse and die of the disease.^3^, ^4^ Despite recent progress in targeted molecular therapies (anti‐*FLT3*, anti‐*IDH1*, *IDH2* or anti‐*Bcl2*), AML treatment has remained essentially the same for 30 years and is still based on variants of the classic “backbone 3 + 7”, associating anthracycline and cytarabine, especially in young and/or fit AML patients. Thus, in 2024, AML is still a major unmet medical need, and the prognosis remains poor.^5^

One aspect of leukaemic cell resistance to chemotherapy is the development of clones with increased capacity to respond to cellular stress. Exposure to chemotherapy or any cytotoxic compounds tends to select clones that develops efficient cell stress mechanisms, which often act by accelerating the removal of mediators of cells death.^6^, ^7^

In this context, AML clone that resist to chemotherapy are expected to have among other systems, an increased expression of enzymes that detoxify reactive aldehyde molecules that induce cell death unless there are inactivated. The aldehydes dehydrogenases (ALDHs) enzymes family contains at least 19 isoforms that impact the redox homeostasis and the production of reactive oxygen species (ROS) by metabolising endogenous and exogenous aldehydes.^8^, ^9^ Recently, ALDH1A1 gene expression was shown to associate to leukaemic stemness pattern and disease outcome: lower ALDH1A1 expression exhibits a more favourable prognosis, in opposition ALDH1A1 and ALDH2 were described as potential actionable targets due to their association with bad risk group classification and survival.^10^

ROS have many functions in biological systems, such as the peroxidation of unsaturated lipids, leading to the intracellular accumulation of highly apoptogenic aldehydes. Among these different apoptogenic aldehydes, two are particularly relevant: the aldehyde malondialdehyde (MDA), which is a chromatin crosslinking agent, and the 4‐hydroxynonenal (HNE), which induces directly cellular apoptosis. HNE affect the cellular redox status by depleting glutathione (GSH); thus, GSH depletion induces a mitochondrial crisis with ROS production and activation of caspase‐mediated apoptosis pathway. The different isoforms of ALDHs, especially ALDH1 (ALDH1A1, 1A2 and 1A3), control the levels of MDA and HNE. Therefore, through the modulation of intracellular HNE concentration, ALDHs control this GSH‐based antioxidant redox system (GRS) and the cellular redox status. Cancer cells protect themselves from the apoptogenic effect of these aldehydes by the ALDHs that oxidise them to non‐apoptogenic carboxylic acids.^9^, ^11^

Drugs that have the ability to inhibit this enzyme are potential candidates for AML treatment. Dimethylthioampal (DIMATE) is an active enzyme‐dependent, competitive and irreversible inhibitor of ALDHs 1 and 3. By inhibiting those ALDHs, DIMATE induces accumulation of apoptogenic aldehydes and disruption of redox homeostasis through a unregulated accumulation of ROS leading to cytotoxic effect.^12^ Cancer cells are more dependent of these antioxidant mechanisms for their survival and consequently more vulnerable to compounds that suppress key antioxidant systems.^13^ Inducing preferential death on cancer cells based on a differential redox state in normal and malignant cells appears to be a new therapeutic approach, as demonstrated by the in vitro and in vivo cytotoxic activity of DIMATE on both LSC and mature blasts while sparing healthy haematopoietic stem cells.^14^

The different ALDHs in human leukaemic cells mediate resistance to a number of drugs, and elevated levels of ALDH activity predict for a poor response to treatment and outcome. Many conventional chemotherapies used for the treatment of AML, such as cytarabine, lead, on the one hand, to an increase of intracellular level of ROS and lipid peroxidation (LP), but also, on the other hand, of ALDH. In parallel, cancer cells rapidly develop drug resistance through a redox adaptation of their metabolism.^15^

Through this work, we will focus on underlying, in 93 AML patients, the correlation between ALDH1A1/2 activity and leukaemic cells redox status, with the ELN 2002 classification at diagnosis, the therapeutic response and overall survival. Then, we will study in vitro short‐term drug responsiveness through a redox blast profile analysis to underpin the potential of using ALDH inhibitor in the AML treatment algorithm.

## 2 PATIENTS AND METHODS

### 2.1 Prospective cohort of AML patients and patients' samples

From September 2015 to April 2019, 93 new AML patients (university hospitals of Marseille, France) were enrolled after informed consent. Inclusion criteria included diagnosis of de novo AML, therapy‐related AML and secondary AML to myelodysplastic syndrome or myeloproliferative neoplasm, except for acute promyelocytic leukaemia (APL). Patients had to have ≥20% blasts in the blood or bone marrow (BM). At diagnosis, before the first line of treatment, BM samples were collected from the 93 (100%) included patients. Among these 93 patients, 59 (64.1%) BM samples were taken before the first line treatment, either after the first consolidation by high dose or intermediate dose of cytarabine or after 4 cycles of treatment with demethylating agents (Azacytidine, AZA). CR was defined by the presence of <5% blasts in the BM, with >1 × 10^9^/L neutrophils and >E100 × 10^9^/L platelets in the PB with no detectable extramedullary disease.^5^ Patients who met the above criteria but had neutrophil or platelet counts less than the stated values were considered to have achieved CRi (CR with incomplete recovery of PB counts). Prognosis stratification was evaluated according to ELN 2022 classification (ELN 2022). Patients and samples' characteristics are summarized in the Table 1.

**TABLE 1:** AML patients and samples' characteristics.

Patient *n* (%)	93 (100%)
Sex *n* (%)	
Male	56 (60.2%)
Female	37 (39.7%)
Median age at diagnosis (range)	72 (range 24–93)
ELN 2022 prognostic groups *n* (%)	
Favourable	15 (16.31%)
Intermediate	34 (36.7%)
Adverse	37 (40.2%)
Unknown	7 (7.6%)
AML subtype *n* (%)	
de novo	48 (51.6%)
Therapy related	5 (5.4%)
Post MDS	19 (20.4%)
Post MPN	21 (22.6%)
First‐line treatment *n* (%)	
High dose of chemotherapy^a^	30 (32.3%)
Azacytidine	50 (53.8%)
Low dose of cytarabine	5 (5.4%)
Best supportive care	8 (8,6%)

^a^ Conventional induction with 3 days of anthracycline and 7 days of cytarabine, followed by 1 to 3 cycles of high dose or intermediate dose of cytarabine.

### 2.2 Bioinformatics analysis

Bioinformatic tools were used to analyse the impact of ALDHs in AML processes: the 165 AML samples of The Cancer Genome Atlas (TCGA) PanCancer Atlas data were used as confirmation cohort of data sample.^16^

### 2.3 Gating Strategy

Red blood cells were first lysed using NH4Cl solution, and the remaining cells were sequentially centrifuged (600 G) for 10 min, washed twice in phosphate buffered saline (PBS: pH 7.2) and resuspended in PBS. BM samples were diluted in order to obtain 106 cells in 100 μL. Then, white blood cells were incubated with appropriate antibodies. Cells were analysed with a Navios (Beckman Coulter) flow cytometer and Kaluza Analysis software v1.5a (Beckman Coulter). Cell populations were gated using forward scatter (FCS), side scatter (SSC) and CD45 or CD34 fluorescence. Briefly, CD45/SSC gating clearly separates mature and immature haematopoietic cell populations in the BM. Lymphocytic cells show the highest CD45 fluorescence intensity and the lowest SSC. Monocytic cells express slightly lower but still high amounts of CD45 and are distinguished from lymphocytes by their higher SSC. Granulocytic cells express low CD45 and broad SSC related to their granulations. Nucleated erythroid cells are characterised by reduced/absent CD45 expression and low SSC; this region can also contain mature (anucleate) red cells and cellular debris, which are eliminated by red blood cell lysis and forward scatter threshold. With this strategy, the earliest cells committed to each lineage occupy a position of low‐medium SSC and CD45 dubbed the “bermudes” area of progenitors.

### 2.4 Flow cytometry quantification of ROS level

White blood cells were incubated with 4 μM of CM‐H2DCFDA (Molecular Probes, Waltham, MA), anti‐CD45KrO (Beckman Coulter, clone J33, 5 μL) and anti‐CD34‐AF750 (Beckman Coulter, clone J33, 5 μL) for 30 min at +37°C. Cells were selected according to CD34 expression and ROS level was quantified on CD34‐positive cells (mean fluorescence intensity [MFI]).

### 2.5 Flow cytometry quantification of ALDH1A1/2 activity

White blood cells were incubated with 5 μM of resorufin propionate (Advanced BioDesign), anti‐CD45KrO (Beckman Coulter, clone J33, 5 μL) and anti‐CD34‐AF750 (Beckman Coulter, clone J33, 5 μL) for 30 min at +37°C. Cells were selected according to CD34 expression, and ALDH1A1/2 activity level was quantified on CD34‐positive cells (mean fluorescence intensity [MFI]) gating strategy.

Briefly, CD45/SSC gating clearly separates mature and immature haematopoietic cell populations in the BM. Lymphocytic cells show the highest CD45 fluorescence intensity and the lowest SSC. Monocytic cells express slightly lower but still high amounts of CD45 and are distinguished from lymphocytes by their higher SSC. Granulocytic cells express low CD45 and broad SSC related to their granulations. Nucleated erythroid cells are characterised by reduced/absent CD45 expression and low SSC; this region can also contain mature (anucleate) red cells and cellular debris, which are eliminated by red blood cell lysis and forward scatter threshold. With this strategy, the earliest cells committed to each lineage occupy a position of low‐medium SSC and CD45, dubbed the “bermudes” area of progenitors.

### 2.6 Flow cytometry quantification of ROS level

After a blood cell lysis of BM samples, white blood cells were incubated with 4 μM of CM‐H2DCFDA (Molecular Probes, Waltham, MA), anti‐CD45KrO (Beckman Coulter) and anti‐CD34‐AF750 (BD Biosciences) for 30 min at +37°C. Cells were analysed with a Navios (Beckman Coulter) flow cytometer and Kaluza Analysis software v1.5a (Beckman Coulter). Cell populations were gated using forward scatter (FCS), side scatter (SSC) and CD45 or CD34 fluorescence.

### 2.7 Flow cytometry quantification of ALDH1A1/2 activity

After a blood cell lysis of BM samples, white blood cells were incubated with 5 μM of resorufin propionate (Advanced BioDesign), anti‐CD45KrO (Beckman Coulter) and anti‐CD34‐AF750 (BD Biosciences) for 30 min at +37°C. Cells were analysed with a Navios (Beckman Coulter) flow cytometer and Kaluza Analysis software v1.5a (Beckman Coulter). Cell populations were gated using forward scatter (FCS), side scatter (SSC) and CD45 or CD34 fluorescence.^17^

### 2.8 Cell sorting

BM were isolated by Ficoll‐Hystopaque density gradient centrifugation (Sigma‐Aldrich, Saint‐Quentin, France). CD34^+^ cell sorting was first performed with CD34 MicroBead Kit UltraPure (MACS; Miltenyi Biotec). Then leukaemic and healthy CD34^+^CD38^−^ or CD34^+^CD38^+^ subpopulations were obtained by flow cytometry cell sorting using double staining with anti‐CD34 (APC MACS; Miltenyi Biotec) and anti‐CD38 (FITC, MACS; Miltenyi Biotec) mAbs, with an exclusion of at least 20 channels between the CD38^+^ and C38^−^ subpopulations (data not shown). The purity of the preparation (≥99% of CD34^+^CD38^−^ leukaemic or healthy cells) was assessed by flow cytometry reanalysis of sorted cells. CD34^+^CD38^−^ enriched cells were plated during 48 h at 6.10^4^ cells/ml in CellGro GMP SCGM medium supplemented with rh SCF (100 ng/mL), rh TPO (20 ng/mL), and rh Flt3 (50 ng/mL) (all from CellGenix GmbH, Freiburg, Germany).

### 2.9 Cell viability

Cells were seeded into 96‐well plates at a concentration of 50,000 cells/well and treated with different concentration of DIMATE for 48 h and analysed using resazurin dye (Sigma‐Aldrich) on a TriStar LB 941 Multimode Microplate Reader (Berthold Technologies, Thoiry, France). Drug responses were quantified by the determination of the half maximal inhibitory concentration (IC_50_) for each cell using a nonlinear fourth‐parameter curve.

### 2.10 Statistical study

All statistical analyses were conducted using Graph Prism v10 or JMP v14 software. Values are expressed as mean ± sem., median or frequencies. Difference comparisons were made according to the normal law when it applied or non‐parametric tests when it was not the case (unpaired *t*‐test), Ordinary one‐way ANOVA Tukey's multiple comparison test. Survival was calculated using the Kaplan–Meyer method and Logrank Test. Correlation was performed using Pearson Method. The threshold of significance was <0.05.

## 3 RESULTS

### 3.1 At diagnosis, ROS level and ALDH1A1/2 activity in AML patients BM are correlated with the different ELN 2022 prognostic groups and overall survival

**FIGURE 1:**
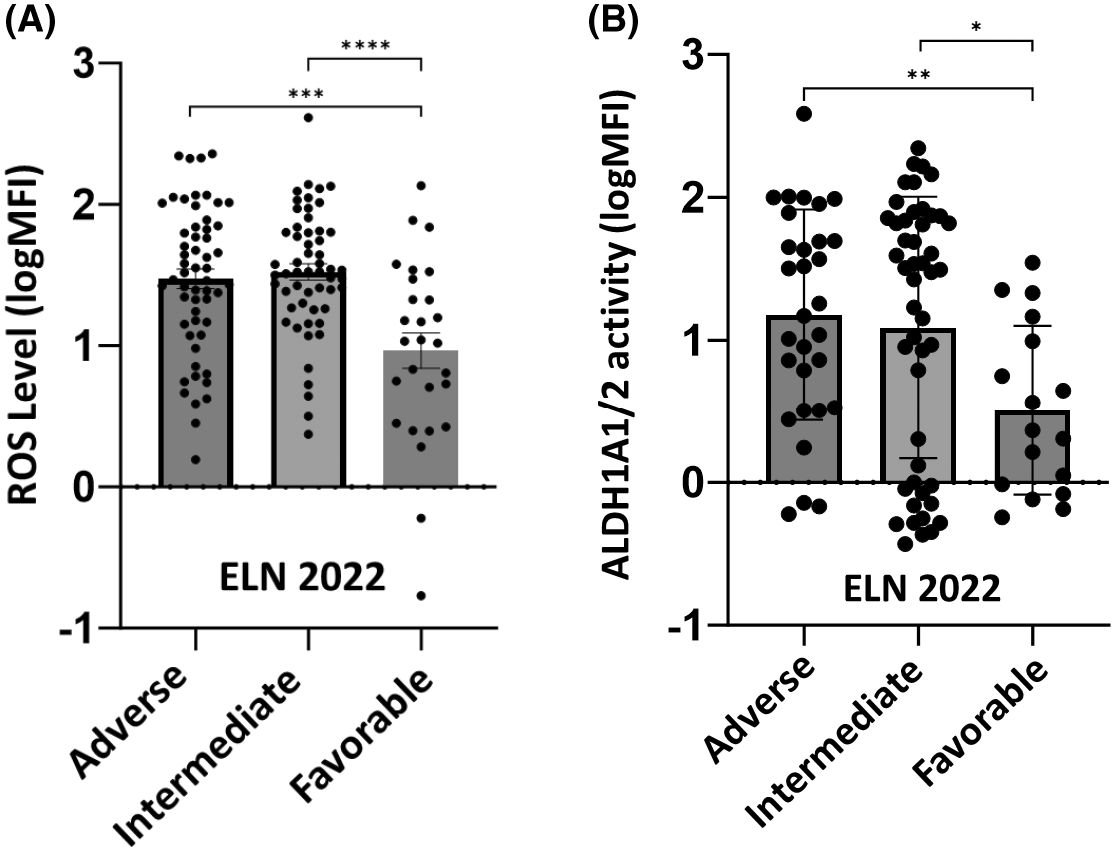
ROS and ALDH1A1/2 mean MFI level in BM at diagnosis, according the different ELN 2022 prognostic subgroups in 93 AML patients at diagnosis. (A) ROS level in BM according the different ELN 2022 prognostic subgroups: ROS level was significantly lower in the favourable ELN 2022 subgroup (*n* = 15) (mean logMFI at 0.964, SEM ± 0.122) compared to the intermediate group (*n* = 34) (mean logMFI at 1.522, SEM ± 0.433, ****p < 0.0001) and the adverse group (*n* = 37) (mean logMFI at 1.473, SEM ± 0.518, ***p < 0.0002) (B) ALDH1A1/2 activity in BM according the different ELN 2022 prognostic subgroups: There was a significant lower activity level in the favourable ELN2022 subgroup (mean logMFI at 0.508, SEM ± 0.198) compared to the intermediate (mean logMFI at 1.088, SEM ± 0.306, *p = 0.0184) and adverse group (mean logMFI at 1.178, SEM ± 0.246, **p = 0.0026).

ROS and ALDH1A1/2 levels have been assessed by flow cytometry at diagnosis on 93 AML patients in BM (Figure 1). The ROS levels were significantly lower in the favourable ELN 2022 subgroup (mean logMFI 0.964 ± 0.122) compared to the intermediate group (mean logMFI 1.522 ± 0.433, *p* < 0.0001) and the adverse group (mean logMFI 1.473 ± 0.518, *p* < 0.0002) (Figure 1A). There was no significant difference in ROS level between the ELN 2022 intermediate and adverse prognostic groups. Regarding ALDH1A1/2 activity in BM, a significant lower activity level in the favourable ELN2022 subgroup (mean logMFI 0.508 ± 0.198) was observed compared to the intermediate (mean logMFI 1.088 ± 0.306, *p* < 0.05) and the adverse group (mean logMFI 1.178 ± 0.246, *p* < 0.01) (Figure 1B). There was no significant difference in ALDH1A1/2 activity between the ELN 2022 intermediate and adverse prognostic groups.

**FIGURE 2:**
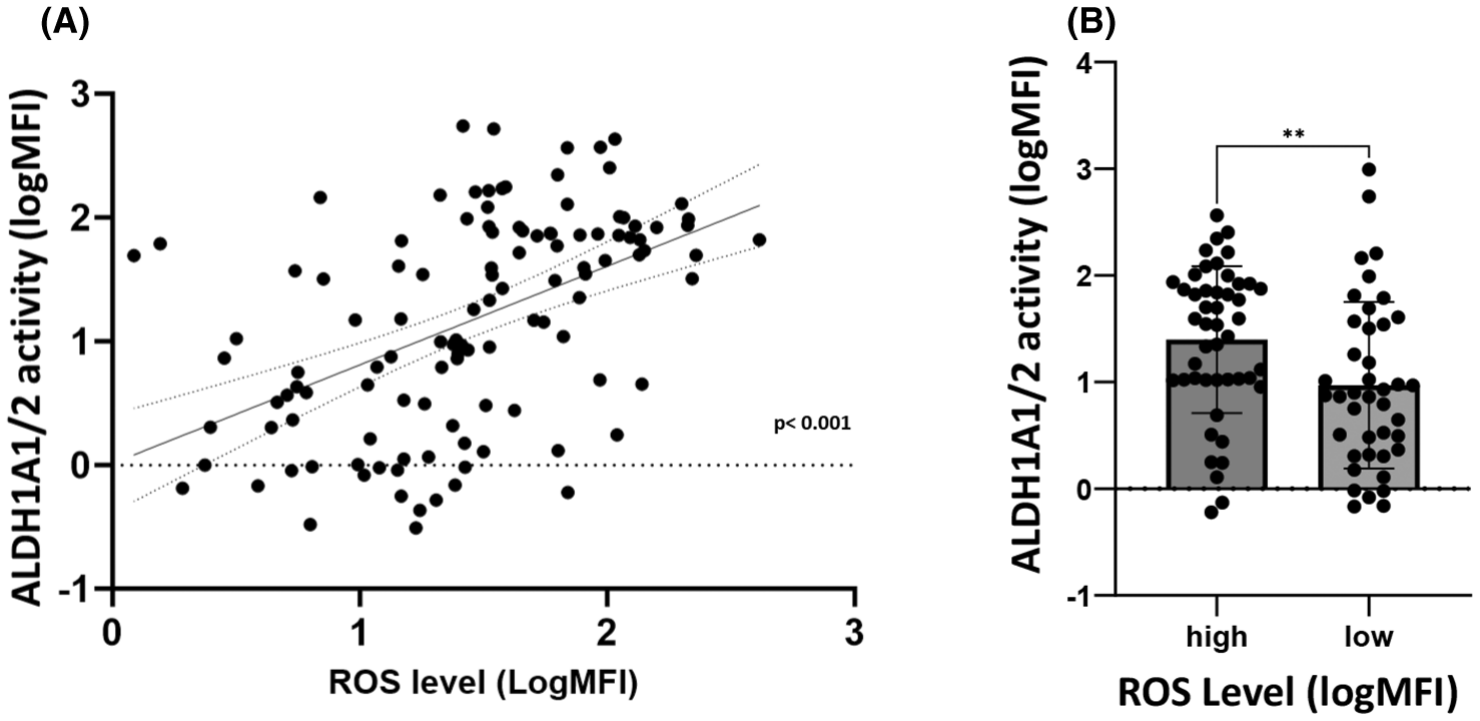
Correlation between ROS level and ALDH1A1/2 activity (in mean MFI) at diagnosis in 93 AML patients. (A) Correlation between the ALDH1A1/2 activity and ROS level (in logMFI) in BM leukaemic blasts at diagnosis in 93 AML patients. There is a strong and significant correlation between ALDH1A1/2 activity and ROS levels (Pearson *r* = 0.4952; *p* < 0.001) (B) ALDH1A1/2 activity in BM according the ROS level status (high vs. low): ALDH1A/2 mean activity is significantly higher in the 40 AML patients considered as ROS^high^ (mean logMFI at 0.97, SEM ± 0.1) compared to the 53 AML patients considered as ROS^low^ (mean logMFI at 0.508, SEM ± 0.122, *p* = 0.0076).

**FIGURE 3:**
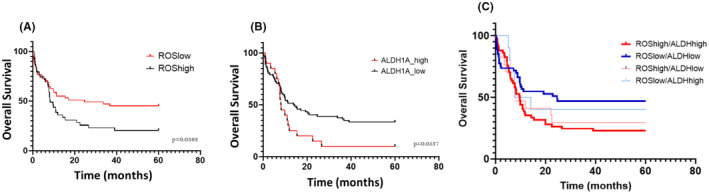
Correlation between ROS status (low vs. high), ALDH1A1/2 status (low vs. high) at diagnosis in 93 AML patients and overall survival (OS). (A) Correlation between ROS status at diagnosis (low vs. high) and OS: ROS^high^ AML patients had a significant lower median overall survival (OS) (8.2 months) than ROS^low^ patients (24.6 months) (HR 3.004, CI 95%, 1.802 to 5.008, *p* = 0.0368). (B) Correlation between ALDH1A1/2 status (high vs. low) and OS: ALDH1A1/2 ^high^ AML patients had a lower median OS (8.13 months) than ALDH1A1/2 ^low^ patients (14 months) (HR 1.723, CI 95%, 1.003 to 2.962). This difference is not statistically significant, but there is a pronounced trend (*p* = 0.0537). (C) Correlation between ROS status and ALDH1/2 at diagnosis (low vs. high) and OS: ROS^high^/ALDH^high^ AML patients had a significant lower median overall survival (OS) (9.7 months) than ROS^low^/ALDH^low^ patients (24.6 months) (HR 1.69, CI 95% 1.064 to 2.681, *p* < 0.05). The difference in OS between ROS^high^/ALDHl^low^ patients (8.2 months) and ROS^low^/ALDH^high^ patients (10.9 months were not significant).

A significant correlation was observed between ALDH1A1/2 activity and ROS level (*ρ* = 0.49; *p* < 0.001) (Figure 2A). The ALDH1A/2 mean activity was significantly higher in the 40 AML patients considered as ROS^high^ (mean logMFI 0.97 ± 0.1) compared to the 53 AML patients considered as ROS^low^ (mean logMFI 0.508 ± 0.122, *p* = 0.0076) (Figure 2B). In addition, ROS^high^ AML patients had a significantly lower median overall survival (OS) (8.2 months) than ROS^low^ patients (24.6 months) (HR 3.004, CI 95%, 1.802 to 5.008, *p* = 0.0368) (Figure 3A). In the same way, ALDH1A1/2^high^ AML patients had a lower median OS (8.13 months) than ALDH1A1/2^low^ patients (14 months) (HR 1.723, CI 95%, 1.003 to 2.962). These differences were not statistically significant, but there is a strong trend (*p* = 0.0537) (Figure 3B). ROS^high^/ALDH^high^ AML patients had a significantly lower median overall survival (OS) (9.7 months) than ROS^low^/ALDH^low^ patients (24.6 months) (HR 1.69, CI 95% 1.064 to 2.681, *p* < 0.05). The difference in OS between ROS^high^/ALDH^low^ patients (8.2 months) and ROS^low^/ALDH^high^ patients (10.9 months) was not significant (Figure 3C).

### 3.2 After first line therapy, an increase of ROS and ALDH1A1/2 level in leukaemic blasts of AML patients were observed, especially in the refractory ones

**FIGURE 4:**
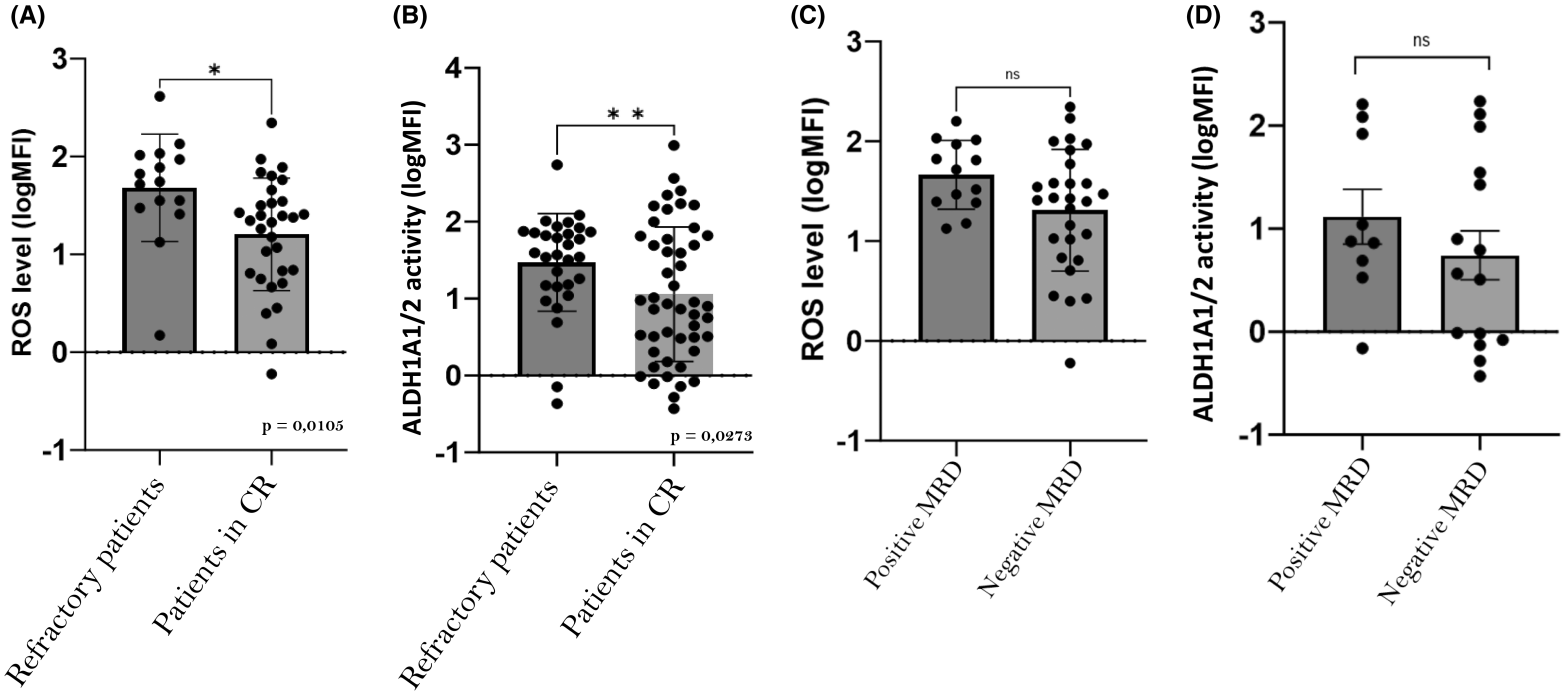
Correlation in 59 AML patients between the therapeutic response after the first line therapy (refractory vs. complete remission (CR)) and mimimal residual disease (MRD) (in 24 patients in complete remission (CR)) with ROS level (mean logMFI) and ALDH1A1/2 activity (mean logMFI) (A) Correlation between ROS level (mean logMFI) and the therapeutic response after the first line therapy (refractory vs. CR): Refractory patients (*n* = 15) had a significant higher mean ROS level in logMFI (1.681, SEM ± 0.547) than patients in CR (*n* = 44) (1.205, SEM ± 0.574), *p = 0.0105 (B) Correlation between ALD1A1/2 activity (mean logMFI) and the therapeutic response after the first line therapy (refractory vs. CR): Refractory patients (*n* = 15) had a significant higher ALDH1A1/2 activity in logMFI (1470, SEM ± 0.635) than patients in CR (*n* = 44) (1.055, SEM ± 0.875), *p = 0.0273. (C) Correlation between ROS level and MRD status (positive vs. negative): Patients with positive MRD (*n* = 9) had higher ROS level (mean logMFI at 1.559, SEM ± 0.444) than patients with negative MRD (*n* = 15) (mean logMFI at 1.279, SEM ± 0.0625). This difference was not statistically significant (*p* = 0.42) (D) Correlation between ALDH1A1/2 activity and MRD status (positive vs. negative): Patients with positive (*n* = 9) MRD had higher ALDH1A1/2 level (mean logMFI at 1.115, SEM ± 0.795) than patients with negative MRD (mean logMFI at 0.7405, SEM ± 0.922). This difference was not statistically significant (*p* = 0.322).

ROS and ALDH1A level in BM were also assessed by flow cytometry in 59 patients after the first line therapy (either after the first consolidation by high dose or intermediate dose of cytarabine or after 4 cycles of treatment with AZA). ROS level was significantly higher in the 15 refractory patients (mean MFI 1.681 ± 0.547) compared to the 44 AML patients in CR (mean logMFI 1.205 ± 0.574) (*p* = 0.015) (Figure 4A). In the same way, a significant difference was observed in ALHD1A1/2 activity between refractory patients (mean logMFI 1.470 ± 0.635) and patients in CR (mean logMFI 1.055 ± 0.875) (*p* < 0.0273) (Figure 4B). For 24 of the 44 patients in CR, minimal residual disease (MRD) was assessed by flow cytometry (Figure 4C,D). Patients with positive MRD (*n* = 9) had higher ROS level (mean logMFI 1.559 ± 0.444) than patients with negative MRD (*n* = 15) (mean logMFI 1.279 ± 0.0625), suggesting higher ROS levels in residual leukaemic blast than in normal blasts. However, the difference was not statistically significant (*p* = 0.42) (Figure 4C). In addition, patients with positive MRD had higher ALDH1A level (mean logMFI 1.115 ± 0.795) than patients with negative MRD (mean logMFI 0.7405 ± 0.922). (Figure 4D), however, this difference were not statistically significant (*p* = 0.322).

**FIGURE 5:**
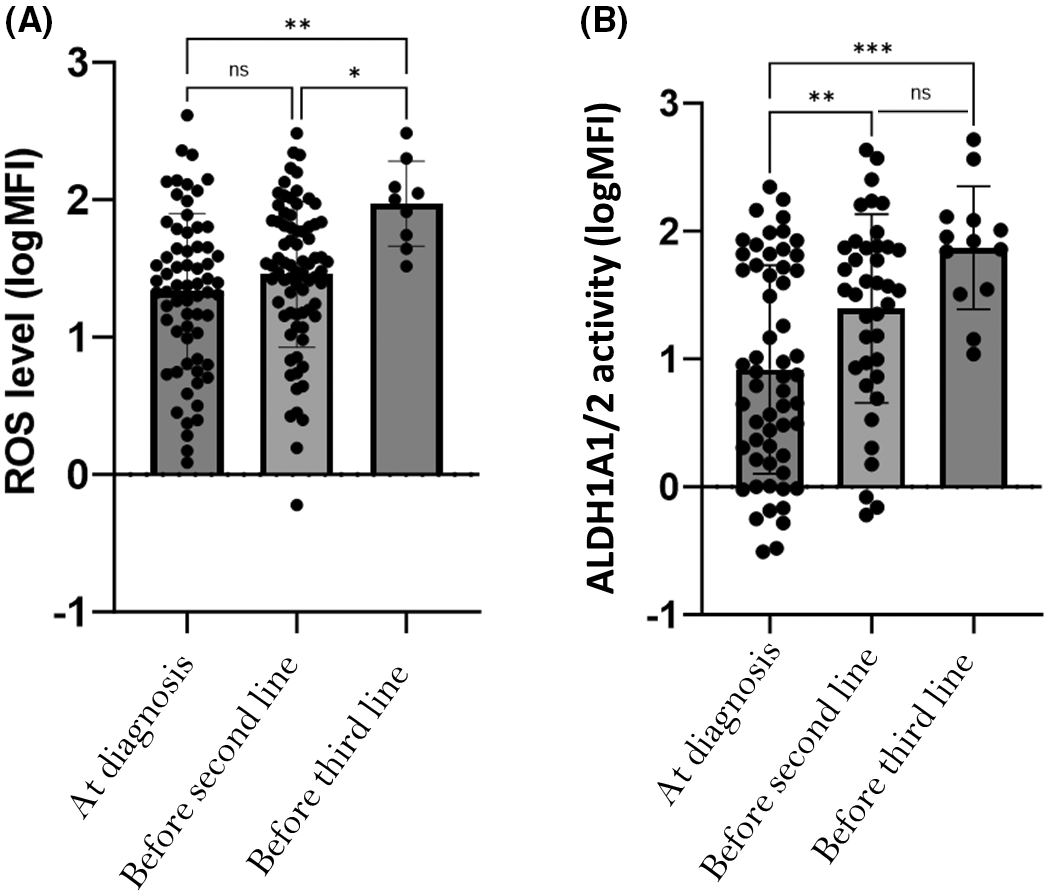
Correlation between ROS level and ALDH1A1/2 activity with the different lines of treatment. (A) Correlation between ROS level with the different lines of treatment: ROS level in BM is enhancing before each line of treatment. ROS mean level was lower at diagnosis than (mean logMFI at 1.333 ± 0.067) (*n* = 93) in relapsed/refractory patients (before the second line of treatment) (*n* = 66) (mean logMFI at 1.457 ± 0.53) (ns). Before the third line of treatment, ROS mean level was significantly higher (mean logMFI at 1.791 ± 0.1) (*n* = 9) compared to diagnosis (**p < 0.001) and after the second line of treatment (*p < 0.05). (B) Correlation between ALDH1A1/2 activity with the different lines of treatment: ALDH1A1/2 mean activity in BM is enhancing before each line of treatment. ALDH1A1/2 mean activity was lower at diagnosis (mean logMFI at 0,917 ± 0.11) (*n* = 93), than in relapsed/refractory patients (before the second line of treatment) (*n* = 66) (mean logMFI at 1.39 ± 0.12) (**p < 0.01). Before the third line of treatment, ALDH1A1/2 mean activity was significantly higher (mean logMFI at 1.869 ± 0.48) (*n* = 12) compared to diagnostic (***p < 0.001). The difference before the second and the third line of treatment is not statistically significant.

During the different lines of treatment, an increase of ROS level and ALDH1A1/2 activity were noticed in refractory and/or relapsed AML patients (Figure 5). ROS level in BM increased between each line of treatment. ROS mean level was lower at diagnosis (mean logMFI 1.333 ± 0.07), than in relapsed/refractory patients (before the second line of treatment) (mean logMFI 1.457 ± 0.06) (ns). Before the third line of treatment, ROS mean level was significantly higher (mean logMFI 1.791 ± 0.1) (*n* = 9) compared to diagnosis (*p* < 0.001) and after the second line of treatment (*p* < 0.05) (Figure 5A). Same result was observed for ALDH1A1/2 activity, with an enhancement of activity before each line of treatment. At diagnosis, ALDH1A1/2 activity was lower (mean logMFI 0,917 ± 0.11) than in relapsed/refractory patients (before the second line of treatment) (mean logMFI 1.39 ± 0.12) (*p* < 0.01). Before the third line of treatment, ALDH1A1/2 mean activity was significantly higher (mean logMFI 1.869 ± 0.13) compared to diagnostic (*p* < 0.001). The difference before the second and the third line of treatment was not statistically significant (*p* = 0.12) (Figure 5B).

### 3.3 DIMATE inhibits proliferation of patient derived cells in accordance with redox balance

**FIGURE 6:**
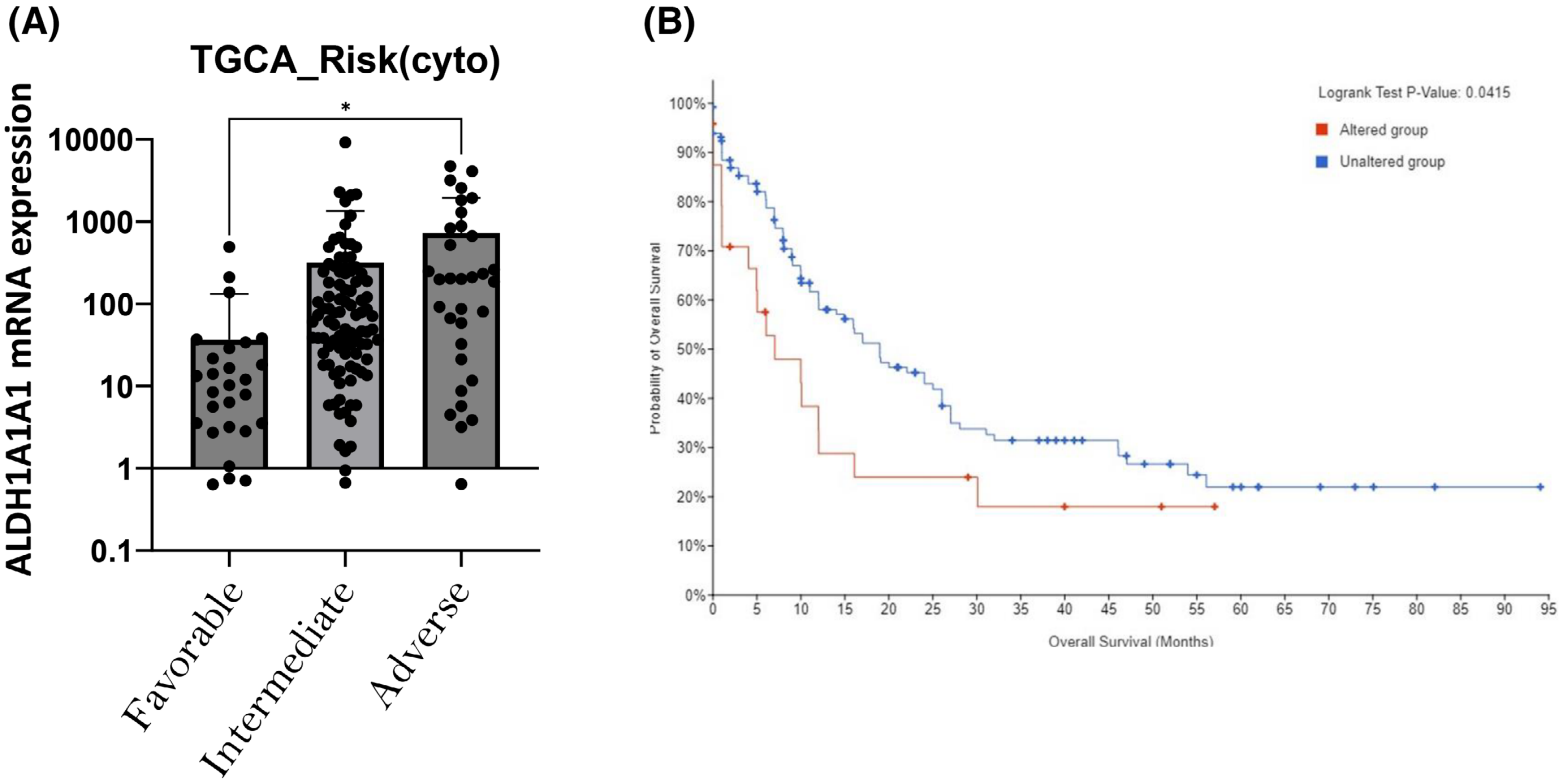
Therapeutic evaluation and correlation of ALDH inhibitor, DIMATE, with ALDH1A1/2 activity and ROS level. (A) Cell cytotoxicity assay performed on Patient Derived cells using DIMATE with a mean IC_50_ equal to 4.46 ± 3.35 μmol/L^−1^ and a median of 2.88 μmol/L. Using a threshold of 25 MFI for ROS level and a threshold of 5 MFI for ALDH1A1/2 activity to distinguish low from high phentotype, ROS level high/ALDH1A1/2 high cells were observed as a significant lower IC_50_ 2.66 ± 1.33 μmol/L and a median 2.68 μmol/L compared to ROS level low/ALDH1A1/2 low cells IC_50_ mean 7.16 ± 3.81 μmol/L^−1^; median 6.85 μmol/L^−1^ (*p < 0.05). (B) Multivariate correlation analysis of DIMATE cells Sensivity, ROS level, ALDH1A1/2 activity and Ratio (ROS level: ALDH1A1/2 activity). Pearson correlation of cell sensitivity were respectively at *ρ* = −0.63 (*p* < 0.05), *ρ* = −0.31 (*p* = 0.45), *ρ* = −0.55 (*p* = 0.16). In addition, a confirmation of correlation, on this subpopulation, were confirmed for ROS level and ALDH1A1/2 activity (*ρ* = 0.69; *p* < 0.05).

Cells from 10 AML patients were tested in vitro using the ALDH inhibitor DIMATE (IC_50_ mean 4.46 ± 3.35 μmol/L^−1^, median 2.88 μmol/L^−1^). Considering that IC_50_ higher than 6.36 μmol/L^−1^ defined a DIMATE resistance phenotype, 80% tested cells were sensitive to DIMATE. Interestingly, cells with both high ROS level and high ALDH1A1/2 activity were significantly more sensitive to DIMATE (IC_50_ = 2.66 ± 1.33 μmol/L; median 2.68 μmol/L; *p* < 0.05) than cells with low ROS levels and ALDH1A1/2 low activity (IC_50_ mean 7.16 ± 3.81 μmol/L^−1^; median 6.85 μmol/L^−1^) (Figure 6A). Based on multivariate correlation analysis comparing ROS level, ALDH1A1/2 activity, ratio of these two parameters and cell sensitivity to DIMATE, ROS level was the strongest predictive factor for the sensitivity of cells to DIMATE with a significant correlation (*ρ* = −0.63; *p* < 0.05), compared to ALDH1A1/2 activity correlation (*ρ* = −0.31; *p* = 0.45) or even ratio ROS level: ALDH1A1/2 activity (*ρ* = −0.55; *p* = 0.16) (Figure 6B).

## 4 DISCUSSION

The poor prognosis of AML patient is due to high relapse rate caused by the flexibility of leukaemic cell genome which can avoid the eradication of all residual diseases. Many explanations were given in order to explain this situation, from the presence of initial quiescent immature LSC to the evolution of resistant leukaemic cells (RCL) who adapt with implementation of mechanism to address circumvention of treatment. Despite this number of explanations, molecular mechanisms of AML resistance are still largely unknown, and new therapy that effectively eradicate leukaemic cells responsible to relapse are an urgent medical need.^2^, ^18^, ^19^

Redox homeostasis may explain this increase of leukaemic cells prone to resist phenotype, and understand the evolution of those biomarker may highlight new therapeutic target and develop new treatment. Since several years ALDH1A emerges as one key member of the ALDH protein family through its role in acute leukaemia.^20^ High RNA expression of ALDH1A was recently shown to be associated with a lower overall survival of AML patients.^9^ In literature, two main paths/pathways have been described from ALDH1A overexpression to AML progression. The first one is the adaptation of leukaemic cells to oxidised lipids, and the second one is the adaptation of AML cells to increased concentrations of retinoic acid due to alterations in their intracellular pathways. However, correlation between ALDH and redox homeostasis was not often mentioned in opposition to glutathione pathway, aldoketoreductase.^21^

On this postulate, in our prospective AML cohort of 93 patients, we have analysed the leukaemic endogenous ROS level and the ALDH1A1/2 activity at different time points of the disease. We demonstrate a strong correlation between ROS and ALDH1A1/2 activity in BM leukaemic blasts suggesting a relationship between a redox state and the ability of the cells to detoxify aldehydes. Then, at diagnosis, ROS level and ALDH1A1/2 activity are correlated to patient's prognosis. Indeed, these two biomarkers correlate with ELN 2022 classification, especially to distinguish between favourable and adverse subgroup. In addition, ROS level, as well as ALDH1A1/2 activity, impact the OS more specifically when the double positive (ROS^high^/ALDH^high^) phenotype is compared with double negative phenotype (ROS^low^/ALDH^low^). These results may appear partially discordant with the results published by Sillar et al.^22^ in 2019, who described a high ROS level in CBF AML, which belongs to the favourable group, and in FLT3 AML patients, which belongs to the intermediate group. In our heterogenous patients cohort, there is a strong proportion of secondary AML and, due to inclusion period, no FLT3 patient was treated with anti FLT3‐ITD inhibitor. We may wonder whether the use of the ELN 2022 classification is appropriate for our work. At diagnosis, there non‐significant difference in ROS level between de novo AML, MRC AML and post‐MPN AML (Figure S1A). Regarding ALDH1A1/2 activity, at diagnosis, there no significant difference between de novo and MRC AML. However, there is a significant difference (*p* < 0.001) between these two subgroups and post‐MPN AML (Figure S1B). In the same way, there no significant difference in ROS level and ALDH1A1/2 activity between the different AML subgroups of interest such as Del17p and/or TP53‐mutated AML or NPM‐1‐mutated AML with the other AML (non‐specific) at diagnosis (Figure S2A,B). Regarding the FLT3 patients issue, only two patients FLT3‐ITD were recruited. One treated by intensive chemotherapy (without Midostaurin, indeed) and one unfit/old patient treated by subcutaneous cytarabine. We do not believe that this significantly alters our results. At last, ELN 2022 stratification still segregate OS in our AML cohort (data not shown).

**FIGURE 7:**
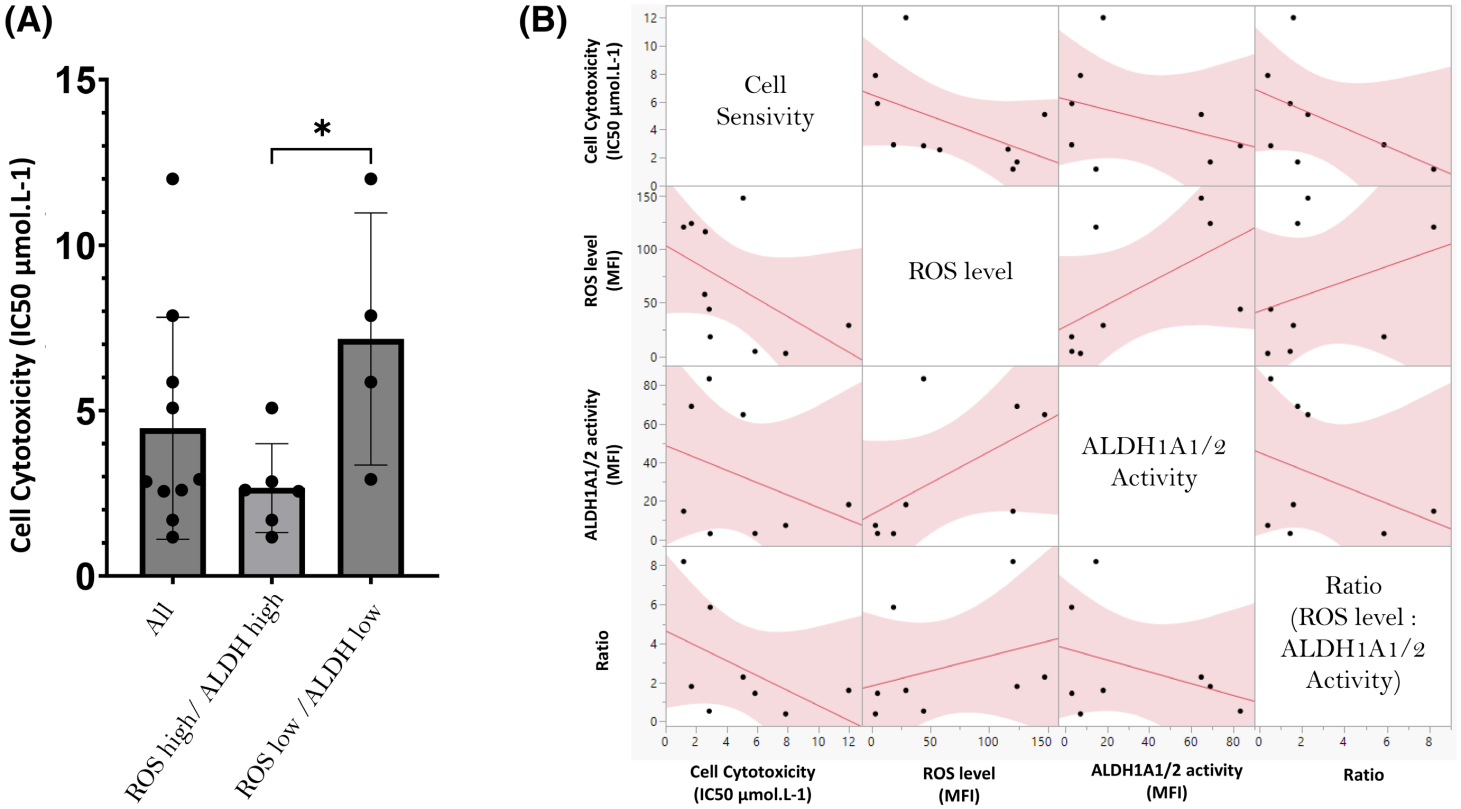
Validation of between molecular risk stratification and median overall survival and ALDH1A1/2 mRNA alteration. (A) ALDH1A1 mRNA expression of TCGA database according the cytogenetic risk favourable, intermediate and adverse. ALDH1A1 mRNA was upregulated in the adverse group (mean 727.8 ± 209.4; *n* = 34) compared to favourable group (mean 36.48 ± 17.06; *n* = 31) (*p* < 0.05) and intermediate group (mean 316 ± 105.6; *n* = 94) (*p* = 0.09). (B) Considering a *z*‐score threshold of 0.5, TCGA population was classified as ALDH1A1/2 altered or unaltered group. A significant lower median overall survival (OS) (median 7.04; CI 95% 4.04 to 30.08) was observed in altered group compared to Unaltered group (median 18.97, CI 95% 12.03 to 26.04) (*p* < 0.005).

Results obtained with our prospective cohort were underpinned by in silico analysis of 165 AML patients RNAseq samples from the TCGA Dataset (TCGA, PanCancer Atlas) Cbioportal assessment of ALDH1A1 mRNA. Indeed, in silico analysis of ALDH1A1 mRNA expression among 165 AML patients in the TCGA Dataset highlighted a significant association between cytologenetic risk and ALDH1A1 mRNA expression level (Figure 7A). Adverse Group mRNA ALDH1A1 expression level (mean 727.8 ± 209.4) was significantly higher than favourable group (mean 36.48 ± 17.06) (*p* < 0.05) but also with intermediate without significance (mean 316 ± 105.6; *p* = 0.09). Based on the same dataset, two ALDH1A1/2 subpopulations could be stratified depending on the *z*‐score relative to diploid sample (*z*‐score threshold <0.5). ALDH1A1/2 altered genetically group (23 mRNA up regulation and 1 deep deletion) representing 15% of all AML patients. Using this stratification, ALDH1A/2 altered patients have a median OS at 7.04 months (CI 95% 4.04 to 30.08), which was significantly lower than ALDH1A/2 unaltered patients with an OS at 18.97 (CI 95% 12.03 to 26.04) (log‐rank *t*‐test *p* < 0.05) (Figure 7B).

Using this cohort, we demonstrate that mRNA expression of ALDH1A1 gene can be used to distinguish adverse from favourable risk and that altered ALDH1A1 gene group (92% with increased ALDH1A1 mRNA), confirming that AML that lacks expression of ALDH1A1 are favourable risk exhibit a lower overall survival rate than unaltered group.^10^ Confirming also previously results that demonstrate that ALDH and more specifically ALDH1A1 may be an actionable targets in AML.^23^ A high expression of ALDH1A1 in AML cells would be a selective advantage in several ways such as resistant to inactivation from byproducts of lipid peroxidation, detoxification of a broad range of aldehyde substrates or facilitating cell survival by regulating critical functions of DNA repair.^24^ That is why ALDH1A1 will enable cells survival after significant exposure to elevated level of oxidant stress and free radicals. Increased level of apoptogenic aldehydes such as methional, malondialdehyde (MDA) and 4‐hydroxynonenal (HNE) can cause damages and senescence in bonne marrow microenvironment and could lead to senescence of normal progenitor, conferring an advantage to ALDH1A1‐overexpressing AML, which will survive in switching from their inflammatory state to reactivate DNA repair.^11^ Leukaemic cells resistant to conventional chemotherapy show a disturbed response to oxidant stress, which provide an advantage to subclones that overexpress ALDH1A1 and explain the association between increased ALDH1A RNA expression and poor prognosis in AML.

From a clinical perspective, this mechanism of action might be relevant. Indeed, as mentioned before ALDH mediate cells refractoriness as well as redox homeostasis. As highlighted by our work, BM PBMC showed an increase of ROS level and ALDH1A1/2 activity after the first line therapy, especially in the refractory patients, whose BM is infiltrated by leukaemic cells. DIMATE has already demonstrated this potential to induce cell death to CD34^+^ leukaemic cells while sparing healthy CD34^+^ progenitors.^14^ To go further, in this work, a demonstration of the efficiency of DIMATE was correlated to the ALDH1A1/2 activity as well as ROS level. In this way, ALDH1A1/2 activity and ROS can therefore be considered as companion tests for predicting the efficacy of DIMATE and selecting patients to whom this drug should be proposed. Moreover, our preliminary results could suggest that DIMATE would be paradoxically more efficient in adverse AML or the refractory ones, since these are the ones with the highest ALDH1A1/2 activity and ROS level. Of course, this in vitro hypothesis will have to be validated in future clinical trials.

In conclusion, all these results indicate that DIMATE could be an alternative in the treatment algorithm of refractory or relapsed AML. It is precisely in this indication that DIMATE, which is pharmacological form named ABD‐3001, is currently being developed in monotherapy through the “first in human” multicenter clinical trial. The ODYSSEY Phase I trial (NCT05601726) is designed for patients with relapsed high‐risk AML or myelodysplastic syndromes who have no therapeutic alternative. According to the phase I trials results, further trials in combination with drugs such as azacitidine or cytarabine, which lead to an increase of intracellular level of ROS and/or ALDH1A1/2 could be proposed.^12^

## AUTHOR CONTRIBUTIONS

**G. Venton:** Conceptualization (equal); data curation (equal); formal analysis (equal); investigation (equal); methodology (equal); project administration (equal); supervision (equal); validation (equal); writing – original draft (equal). **J. Colle:** Conceptualization (equal); data curation (equal); formal analysis (equal); investigation (equal); methodology (equal); supervision (equal); validation (equal); writing – original draft (equal). **A. Tichadou:** Data curation (equal); investigation (supporting). **J. Quessada:** Data curation (equal); formal analysis (equal). **C. Baier:** Data curation (equal); formal analysis (equal). **Y. Labiad:** Formal analysis (equal); methodology (equal); software (equal). **M. Perez:** Conceptualization (equal); project administration (equal); supervision (equal); validation (equal). **L. De Lassus:** Investigation (equal); validation (equal); writing – original draft (equal). **M. Loosveld:** Formal analysis (equal); software (equal); visualization (equal). **I. Arnoux:** Data curation (equal); formal analysis (equal). **N. Abbou:** Data curation (equal); formal analysis (equal). **I. Ceylan:** Conceptualization (equal); project administration (equal); supervision (equal). **G. Martin:** Conceptualization (equal); data curation (equal); formal analysis (equal); investigation (equal); methodology (equal); project administration (equal); supervision (equal); validation (equal); writing – original draft (equal). **Regis T Costello:** Conceptualization (equal); methodology (equal); project administration (equal); supervision (equal); validation (equal); writing – original draft (equal).

## FUNDING INFORMATION

This study was not funded.

## CONFLICT OF INTEREST STATEMENT

Ismail Ceylan is the CEO of the company ABD, which develops, produces and distributes the ABD‐3001. Guillaume Martin, Yasmine Labiad & Milly Perez are employees of the company ABD. All the other authors declare no competing interest.

## Supporting information


Figure S1.

Figure S2.

## Data Availability

The data that support the findings of this study are available from the corresponding author upon reasonable request.
