# Supplementary figures and images for: Reactive oxygen species and aldehyde dehydrogenase 1A as prognosis and theragnostic biomarker in acute myeloid leukaemia patients
Source: J Cell Mol Med. 2024 Oct 11;28(19):e70011. doi: 10.1111/jcmm.70011 (PMC11467733; doi:10.1111/jcmm.70011)

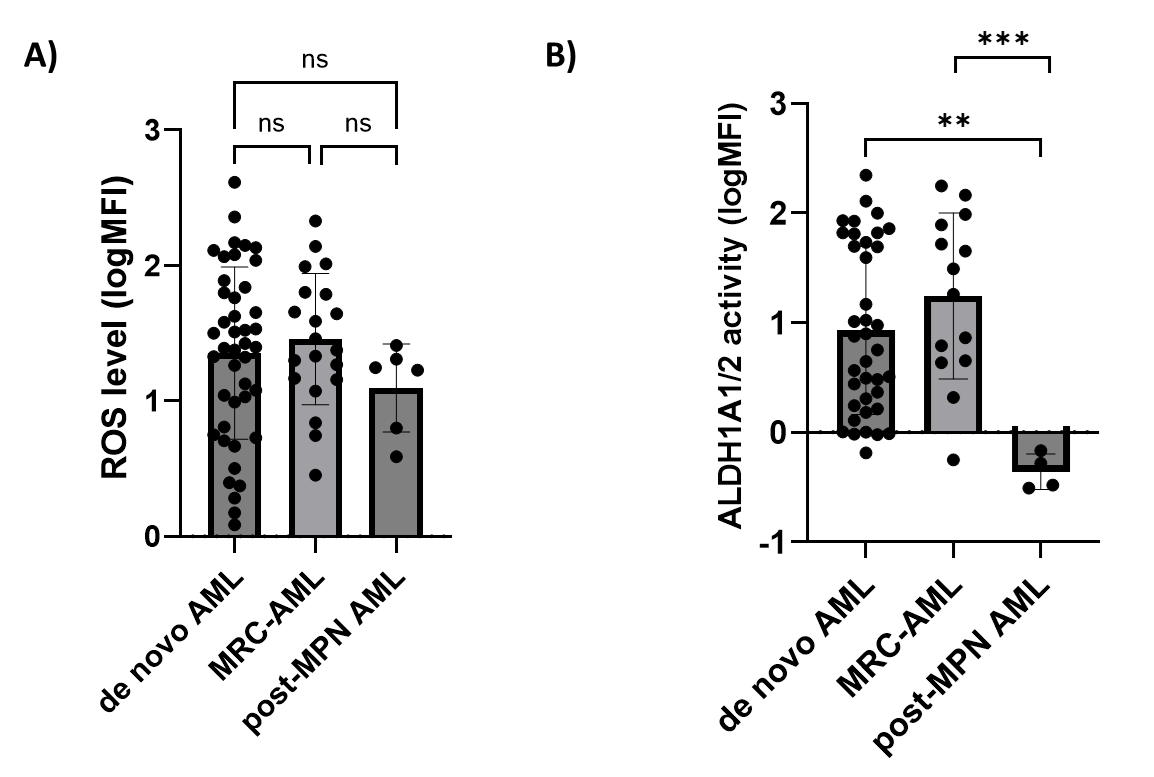

Supplement: Supplementary file 1 — Figure S1. [file JCMM-28-e70011-s001.tif]

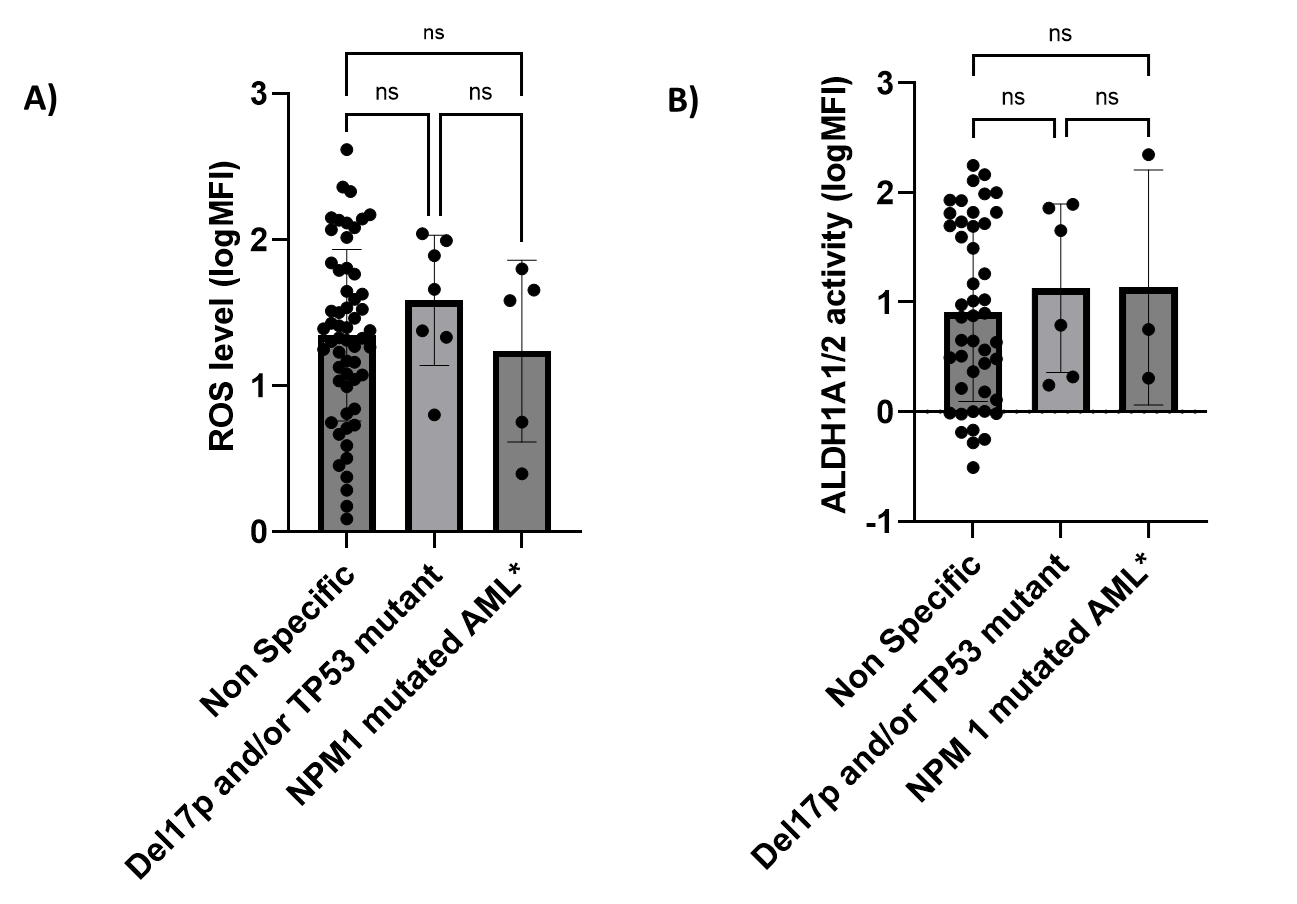

Supplement: Supplementary file 2 — Figure S2. [file JCMM-28-e70011-s002.tif]
